# Maternal Obesity and Tobacco Use Modify the Impact of Genetic Variants on the Occurrence of Conotruncal Heart Defects

**DOI:** 10.1371/journal.pone.0108903

**Published:** 2014-10-02

**Authors:** Xinyu Tang, Todd G. Nick, Mario A. Cleves, Stephen W. Erickson, Ming Li, Jingyun Li, Stewart L. MacLeod, Charlotte A. Hobbs

**Affiliations:** 1 Biostatistics Program, Department of Pediatrics, College of Medicine, University of Arkansas for Medical Sciences, Little Rock, Arkansas, United States of America; 2 Division of Birth Defects Research, Department of Pediatrics, College of Medicine, University of Arkansas for Medical Sciences, Little Rock, Arkansas, United States of America; 3 Department of Biostatistics, College of Public Health, University of Arkansas for Medical Sciences, Little Rock, Arkansas, United States of America; Case Western Reserve University, United States of America

## Abstract

Conotruncal heart defects (CTDs) are among the most severe birth defects worldwide. Studies of CTDs indicate both lifestyle behaviors and genetic variation contribute to the risk of CTDs. Based on a hybrid design using data from 616 case-parental and 1645 control-parental triads recruited for the National Birth Defects Prevention Study between 1997 and 2008, we investigated whether the occurrence of CTDs is associated with interactions between 921 maternal and/or fetal single nucleotide polymorphisms (SNPs) and maternal obesity and tobacco use. The maternal genotypes of the variants in the glutamate-cysteine ligase, catalytic subunit (*GCLC*) gene and the fetal genotypes of the variants in the glutathione S-transferase alpha 3 (*GSTA3*) gene were associated with an elevated risk of CTDs among obese mothers. The risk of delivering infants with CTDs among obese mothers carrying AC genotype for a variant in the *GCLC* gene (rs6458939) was 2.00 times the risk among those carrying CC genotype (95% confidence interval: 1.41, 2.38). The maternal genotypes of several variants in the glutathione-S-transferase (*GST*) family of genes and the fetal genotypes of the variants in the *GCLC* gene interacted with tobacco exposures to increase the risk of CTDs. Our study suggests that the genetic basis underlying susceptibility of the developing heart to the adverse effects of maternal obesity and tobacco use involve both maternal and embryonic genetic variants. These results may provide insights into the underlying pathophysiology of CTDs, and ultimately lead to novel prevention strategies.

## Introduction

Congenital heart defects (CHDs) are among the most common and severe birth defects worldwide, with reported estimated prevalence of 9.1 per 1,000 live births after 1995 [1]. Conotruncal heart defects (CTDs), a class of CHDs, affect the cardiac outflow tracts and great arteries which include truncus ateriosus, interrupted aortic arch type B, transposition of great arteries, double outlet right ventricle, conoventricular septal defect, tetralogy of Fallot, and pulmonary atresia with ventricular septal defect.

Nonsyndromic CTDs are due to a multifactorial etiology involving a complex interplay between genetic susceptibilities and environmental factors [2], [3]. One such interplay is between maternal folic acid supplement use and genetic variants in folate-related pathways. Based on the finding that maternal periconceptional folic acid intake decreases the occurrence of CTDs [4], [5], multiple studies have investigated associations between CTDs and polymorphisms in folate-related genes [6]–[8]. It has also been reported that genetic variants in folate-related pathways modify the association between birth defects and maternal intake of folic acid containing supplements [9].

Developmental toxicology studies using animal models have repeatedly demonstrated the unquestioned importance of genetic variation in determining risks to environmental factors [10]. Inbred strains of mice, representing different mouse genomes, vary in their susceptibility to teratogenic and xenobiotic agents. In reproductive age women, obesity and tobacco use have been associated with multiple adverse outcomes including intrauterine growth retardation [11], [12], prematurity [13], [14], and birth defects [15]–[17]. Obesity and cigarette smoking are also associated with alterations in folate and glutathione metabolism resulting in decreased folate [18], [19], increased homocysteine [20]–[24], and decreased glutathione [25]–[30] that may compromise the *in-utero* environment. Some studies have demonstrated that maternal genetic variants modulate the association between pregnancy smoking exposure and fetal growth restriction [31], [32]. It is possible that variants in genes that encode for critical enzymes in folate, homocysteine and glutathione pathways modify the adverse impact of obesity and tobacco on the developing heart.

In this study, we used a hybrid design which combines genetic and lifestyle data from case-parental and control-parental triads to investigate whether CTDs are associated with interactions between maternal and/or fetal single nucleotide polymorphisms (SNPs) and maternal obesity and tobacco use. In contrast to most published reports of Gene × Environment (G × E) interactions [4], [33], [34], we have evaluated maternal and fetal genetic effects simultaneously. A total of 1536 SNPs in 62 target genes were selected for this study from folate-related metabolic pathways.

## Materials and Methods

### Study Population

Families were recruited for the National Birth Defects Prevention Study (NBDPS) with estimated dates of delivery between October 1997 and August 2008 (www.nbdps.org). Detailed information about the NBDPS is outlined in Yoon et al [35]. Families were identified through population-based birth defects surveillance systems in 10 states: Arkansas, California, Iowa, Massachusetts, New Jersey (through 2002), New York, Texas, Georgia, North Carolina (beginning 2003), and Utah (beginning 2003). In this study, cases were singleton live-born infants with CTDs. NBDPS cardiac cases were reviewed by pediatric cardiologists using a classification strategy developed by investigators within the NBDPS. This strategy targeted etiologic investigations of CHDs that encourage explicit case definitions and aggregates of defects with a focus on simple, isolated phenotypes and associations [36]. Cases with recognized or strongly suspected monogenic or chromosomal conditions were excluded. Controls were singleton live-born infants without any major structural birth defects [37]. Both case and control mothers completed phone interviews. The study was approved by the University of Arkansas for Medical Sciences' Institutional Review Board and the NBDPS with protocol oversight by the Centers for Disease Control and Prevention Center for Birth Defects and Developmental Disabilities. All of the study subjects gave written informed consent. For minors, informed written consent was obtained from their legal guardian.

### Maternal Interview

Participation in the NBDPS included a one-hour interview with mothers of cases and controls, conducted in English or Spanish, by interviewers using a computer-assisted telephone questionnaire [35]. In this study, we investigated how maternal obesity and tobacco use modify maternal and fetal genetic effects on the risk of CTDs. Obesity, using the Institute of Medicine definition [38], was defined as a body mass index (BMI) ≥30.0 and normal weight between 18.5 and 25.0. Smokers were defined as women who smoked cigarettes during the 3 months after conception and nonsmokers otherwise. Maternal use of folic acid supplements is a known risk factor for the occurrence of CTDs and warranted a separate manuscript [9].

### DNA Collection

After completion of maternal interviews, a buccal cell collection kit was sent to participants to obtain cheek cell samples from case/control and parents. The collection kit included informed consent forms, instructions, $20 money order, materials for completing the specimen collection and prepaid US mail packets for specimen return [35].

### Gene/SNP Selection

As previously described [39], a custom panel of 1536 SNPs in 62 genes involved in folate metabolism was developed jointly between our lab and Illumina. Candidate genes were required to encode an enzyme in one of the candidate metabolic pathways and be expressed in liver and/or heart tissue [40]. For each candidate gene, a maximally informative set of haplotype-tagging SNPs was selected using both linkage disequilibrium statistics and Illumina assay design scores. The custom genotyping panel was devised in 2005–2006. At that time there were two genes called *RFC1* in the commonly used genetic databases. The genotype data presented here are for SNPs in the Replication Factor C (activator 1) 1 (*RFC1*) gene. This gene is an activator of DNA polymerase and is required for DNA synthesis and repair.

### Genotyping and Quality Assessment

Genotyping was conducted on a total of 635 case and 1702 control families using 200 ng of WGA DNA on the Illumina Golden Gate platform [41]. Initial genotype calls were generated using Genome Studio's GenCall, Illumina's proprietary algorithm, with subsequent analysis performed using SNPMClust, a bivariate Gaussian model-based genotype clustering and calling algorithm developed in-house. A total of 297 individuals were removed due to study ineligibility (n = 33), high no-call rates (n = 63), or high rates of Mendelian inconsistency (n = 201). We found that the quality of genotype clustering varied substantially from SNP to SNP, which we attribute to the *in silico* design of the SNP panel based on data from phases I and II of the HapMap project, without the subsequent quality checks that would be applied to a standard commercial SNP panel. While the majority of SNPs exhibited well-segregated genotype clusters, a substantial percentage exhibited poor clustering and/or lower minor allele frequency (MAF) than expected. To ensure high-quality genotypes, we applied stringent quality control measures and excluded SNPs with obviously poor clustering behavior (60 SNPs), no-call rates >10% (328 SNPs), Mendelian error rates >5% (11 SNPs), MAF <5% (204 SNPs), or significant deviation from Hardy-Weinberg Equilibrium in at least one racial group (*p*<10^−4^, 12 SNPs). The final dataset included 4648 individuals, each with 921 SNPs.

### Statistical Methods

Descriptive statistics were summarized for families. Comparisons of maternal characteristics were carried out between case and control families using the two-sample t-tests for continuous variables and chi-square tests for categorical variables. Because both case-parental and control-parental triads were enrolled and genotyped, a hybrid design provided optimal power by using a log-linear model [42]. This model has several advantages including incorporating case and control triads, providing population-based inferences related to maternal and fetal genetic effects, providing evidence related to fetal genetic effects that are robust to population admixture, and allowing inclusion of missing genotype data. To assess Gene × Environment (G × E) interactions, a log-linear model was fitted for each SNP as a function of mating types, an interaction between mating types and a maternal factor (E), case/control status (D), maternal genotype (D × M), fetal genotype (D × C), an interaction for maternal genotype (D × E × M), and an interaction for fetal genotype (D × E × C). The detailed information regarding the model is outlined in Hobbs et al [9]. Based on the log-linear model for counts and assuming a Poisson distribution, the relative risk (RR) (95% CI) of having one copy of the minor allele compared to no copy was estimated for each SNP. Because we assumed multiplicative risk of alleles, the estimated RR (95% CI) of having 2 copies of the minor allele is square of the estimated RR (95% CI) of having 1 copy of the minor allele. We also performed sensitivity analysis by restricting our models to the Caucasian families only. The Caucasian families were selected for the sensitivity analysis because among all the racial groups, we had the largest sample size for Caucasian families. For other racial groups, we did not have power to perform any subgroup analyses.

The Bayesian false-discovery probability (BFDP) [43]–[48] was used to evaluate the chance of false-positive associations using estimates of the RR and corresponding 95% CI obtained from log-linear models. In the results section, we reported associations where BFDP <0.80, which is a commonly used threshold suggested by Wakefield [43] for summary analyses. Patterns of linkage disequilibrium (LD) between significant SNPs in the same gene were constructed to assess the correlations among these SNPs using the parents of control families. Data were analyzed using statistical software LEM [49] for fitting log-linear models, R v3.0.1 (R Foundation for Statistical Computing, Vienna, Austria) for computing descriptive statistics, and BFDPs and HaploView 4.2 [50] for developing LD maps.

## Results

### Study Sample

A total of 616 case families and 1645 control families were included in our analysis. Due to different call rates among SNPs, case and control triads/dyads/monads numbers differ for any given SNP. Maternal demographics and lifestyle factors are summarized in **Table 1**. The case mothers were slightly older than the control mothers [28.3±6.1 vs. 27.5±6.0, p = 0.002]. Case and control mothers did not differ on any of the other demographics and lifestyle characteristics (p>0.05).

**Table 1:** Maternal characteristics for 616 case families and 1,645 control families enrolled in National Birth Defects Prevention Study between 1997 and 2008.

	Case	Control
	(N = 616)	(N = 1,645)
**Age at delivery (years)**	28.3±6.1	27.5±6.0
**Race**		
African American	49 (8%)	143 (9%)
Caucasian	401 (66%)	1,136 (69%)
Hispanic	123 (20%)	285 (17%)
Others	39 (6%)	78 (5%)
Missing information	4	3
**Education**		
<12 years	83 (14%)	217 (13%)
High school degree or equivalent	167 (27%)	413 (25%)
1–3 years of college	173 (28%)	454 (28%)
At least 4 years of college or Bachelor degree	190 (31%)	559 (34%)
Missing information	3	2
**Household income**		
Less than 10 Thousand	94 (16%)	236 (15%)
10 to 30 Thousand	150 (26%)	408 (27%)
30 to 50 Thousand Dollars	118 (20%)	348 (23%)
More than 50 Thousand	217 (37%)	538 (35%)
Missing information	37	115
**Folic acid supplements**		
No	299 (49%)	738 (45%)
Yes^1^	314 (51%)	907 (55%)
Missing information	3	0
**BMI**		
Underweight (BMI <18.5)	31 (5%)	74 (5%)
Normal weight (18.5< = BMI <25)	298 (50%)	880 (55%)
Overweight (25< = BMI <30)	141 (24%)	360 (23%)
Obese (> = 30)	121 (20%)	281 (18%)
Missing information	25	50
**Alcohol consumption**		
No	460 (76%)	1,251 (76%)
Yes^2^	149 (24%)	390 (24%)
Missing information	7	4
**Tobacco use**		
No	498 (81%)	1,356 (82%)
Yes^3^	114 (19%)	288 (18%)
Missing information	4	1

Summary statistics were expressed as mean ± standard deviation for continuous variables, and frequency (percentage) for categorical variables.

1 Folic acid supplements yes is defined as maternal use of folic acid supplements at least two months during the exposure window (i.e. one month prior to conception and two months after conception).

2 Alcohol consumption yes is defined as drinking during the three months after pregnancy.

3 Tobacco use yes is defined as cigarette smoking during the three months after pregnancy.

### Genetic Variants

**Figure 1:**
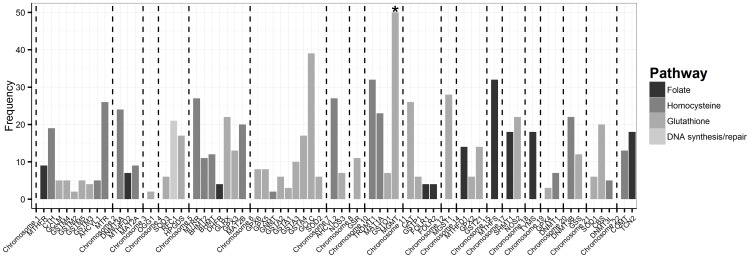
SNP, gene, chromosome and pathway information. Information about the number of SNPs from each gene, chromosome and pathway (* there are a total of 142 candidate SNPs on MGMT, truncated at 50 for display purpose). The average number of SNPs per gene is 15±19. Among 60 genes, 10 (17%) genes are involved in folate pathway, 32 (53%) in glutathione pathway, and 17 (28%) in homocysteine pathway, and one gene RFC1 (2%) in DNA synthetic/repair pathway.

As described above, 921 candidate SNPs were included in the final analyses. The information about the number of SNPs in each gene, chromosome and pathway is displayed in **Figure 1**. Hobbs et al. [9] identified the maternal genotypes of 17 SNPs and the fetal genotypes of 17 SNPs associated with CTD risks independently from interactions with maternal folic acid supplement use, obesity and tobacco use. Briefly, the maternal genotypes of 10 SNPs and the fetal genotypes of 2 SNPs in the glutamate-cysteine ligase, catalytic subunit (*GCLC*) gene were found to be significantly associated with the risk of CTDs through main genetic effects [9].

### Gene × Environment Interactions

In Hobbs et al. [9], we identified SNPs that had a main effect on the occurrence of CTDs. To identify significant G × E interactions, we evaluated the combined effects of each maternal and infant SNP with maternal folic acid supplement use, obesity and tobacco use. Significant interactions are discussed below.

#### SNP × Folic Acid Supplement Use

In Hobbs et al. [9], we have studied the interactions between 921 SNPs and periconceptional folic acid supplement use, and found that the maternal genotypes of 19 SNPs and the fetal genotypes of 9 SNPs had BFDP <0.80 for testing the G × E interactions with maternal use of folic acid supplements. All these SNPs were not found to be significant based on the gene only models. Detailed information about the SNP × folic acid supplement use is outlined in Hobbs et al [9].

#### SNP × Obesity

**Figure 2:**
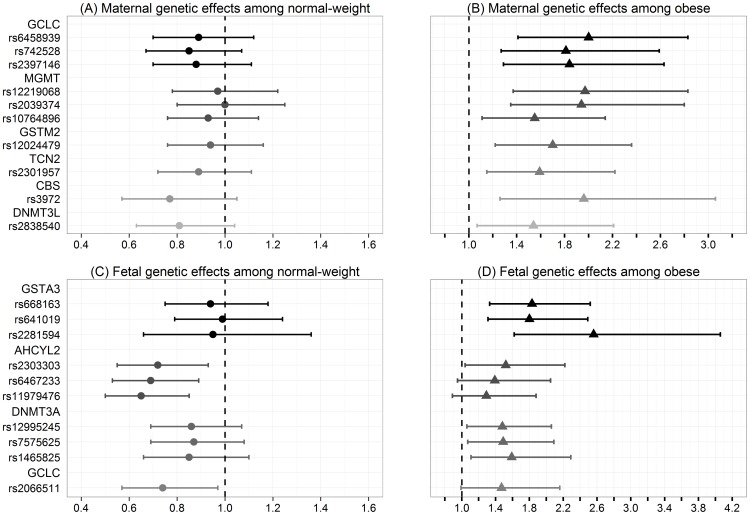
Interactions between gene and obesity. The estimated relative risks and their 95% confidence intervals for each maternal and fetal SNPs that were identified to have a significant interaction with maternal obesity on the risk of CTDs. (A) Maternal genetic effects among normal-weight women; (B) Maternal genetic effects among obese women; (C) Fetal genetic effects among normal-weight women; (D) Fetal genetic effects among obese women.

A main effect of the association between CTDs and obesity compared to normal weight resulted in an estimated RR = 1.27 (95% CI: 0.99, 1.63; p = 0.06). Because previous studies showed that obesity independent of pre-existing Type I or Type II diabetes was significantly associated with elevated risk of CTDs [15], [51], we computed a model for each SNP among normal-weight and obese women. The results are shown in **Figure 2** and **Table S1**.

Among 10 maternal SNPs with a significant gene × obesity interaction, three SNPs are in the *GCLC* gene, three SNPs are in the O-6-methylguanine-DNA methyltransferase (*MGMT*) gene, and the remaining four SNPs are in the glutathione S-transferase mu 2 (*GSTM2*), transcobalamin II (*TCN2*), cystathionine-beta-synthase (*CBS*), and DNA (cytosine-5-)-methyltransferase 3-like (*DNMT3L*) genes respectively. The most significant maternal gene × obesity interaction (i.e. most significant difference in the estimated RR for certain genotype among obese women compared to normal weight women) was embedded in the *GCLC* gene. The genotype of rs6458939 was not associated with the risk of CTDs among normal-weight women [estimated RR: 0.89, 95% CI: 0.70, 1.12]; however, the risk of delivering infants with CTDs among obese women carrying AC genotype for rs6458939 was estimated to be 2.00 (95% CI: 1.41, 2.83) times the risk among those carrying CC genotype. The RR (95% CI) for carrying two copies of the minor allele is square of the estimated RR (95% CI) for carrying one copy of the minor allele. Thus, only the RR (95% CI) is reported for carrying one copy of the minor allele compared to no copy of the minor allele. A similar pattern was observed for the maternal genotypes of two other SNPs in the *GCLC* gene. All three maternal SNPs in the *GCLC* gene were determined to be in high LD (D′≥0.98).

Among 10 fetal SNPs found to be associated with the risk of CTDs in combination with obesity, three SNPs reside in the glutathione S-transferase alpha 3 (*GSTA3*) gene, three SNPs are in the adenosylhomocysteinase-like 2 (*AHCYL2*) gene, three SNPs are in the DNA (cytosine-5-)-methyltransferase 3 alpha (*DNMT3A*) gene, and the other SNP resides in the *GCLC* gene. We observed the most significant difference in the fetal effect of the *GSTA3* gene on the risk of CTDs between normal-weight and obese women. The risk of CTDs was elevated for infants with AG genotype for rs668163 compared to GG genotype among obese women (RR: 1.83, 95% CI: 1.33, 2.52), but was not significantly reduced among normal-weight women (RR: 0.94, 95% CI: 0.75, 1.18). Similar findings were observed for the fetal genotypes of two other SNPs in the *GSTA3* gene. All three fetal SNPs in the *GSTA3* gene were in high LD (D′≥0.96).

Similar results were observed when overweight women were compared to normal-weight women (data not shown) and based on Caucasian families only (data not shown). There was no main effect of any of the SNPs discussed above.

#### SNP × Tobacco Use

**Figure 3:**
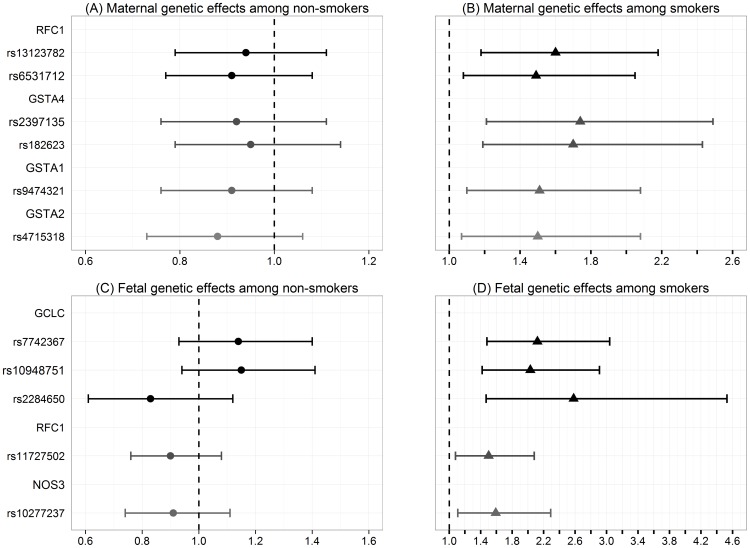
Interactions between gene and maternal tobacco use. The estimated relative risks and their 95% confidence intervals for each maternal and fetal SNPs that were identified to have a significant interaction with maternal tobacco use on the risk of CTDs. (A) Maternal genetic effects among non-smokers; (B) Maternal genetic effects among smokers; (C) Fetal genetic effects among non-smokers; (D) Fetal genetic effects among smokers.

A main effect of the association between CTDs and maternal tobacco use compared to no tobacco use had an estimated RR = 1.08 (95% CI: 0.85, 1.37; p = 0.54). We investigated the interactions between SNPs and maternal tobacco use on the occurrence of CTDs by fitting the G × E log-linear model with maternal tobacco use for each SNP. The maternal and fetal SNPs that had a significant interaction with maternal tobacco use are presented in **Figure 3** and **Table S2**.

Among 6 maternal SNPs found to have BFDP <0.80, two of them are located in the *RFC1* gene, two SNPs are in the glutathione S-transferase alpha 4 (*GSTA4*) gene, and the other two SNPs are in the glutathione S-transferase alpha 1 (*GSTA1*) and glutathione S-transferase alpha 2 (*GSTA2*) genes respectively. The *GSTA1*, *GSTA2* and *GSTA4* genes are glutathione-S-transferase (*GST*) family of genes. The RRs of the maternal AC genotype for rs2397135 in the *GSTA4* gene compared to CC genotype were estimated to be 0.92 (95% CI: 0.76, 1.11) among women without tobacco use, and 1.74 (95% CI: 1.21, 2.49) among women with tobacco use, interaction BFDP = 0.68<0.80. The two maternal SNPs in the *RFC1* gene were in moderate LD (D′ = 0.82).

Among 5 fetal SNPs identified to have BFDP <0.80, three SNPs are in the *GCLC* gene, one SNP (rs11727502) is in the *RFC1* gene, and the other SNP (rs10277237) is located in the nitric oxide synthase 3 (endothelial cell) (*NOS3*) gene. The RRs of the fetal AG genotype for rs7742367 in the *GCLC* gene compared to AA genotype were estimated to be 1.14 (95% CI: 0.83, 1.40) among women without tobacco use, and 2.12 (95% CI: 1.48, 3.04) among women with tobacco use, interaction BFDP = 0.71<0.80. The two fetal SNPs (rs7742367 and rs10948751) in the *GCLC* gene were in high LD (D′ = 0.99), and in moderate LD with the other SNP (rs2284650) respectively (D′ = 0.89 and 0.93).

Similar results were found based on Caucasian families only (data not shown). There was no SNP main effect.

## Discussion

**Figure 4:**
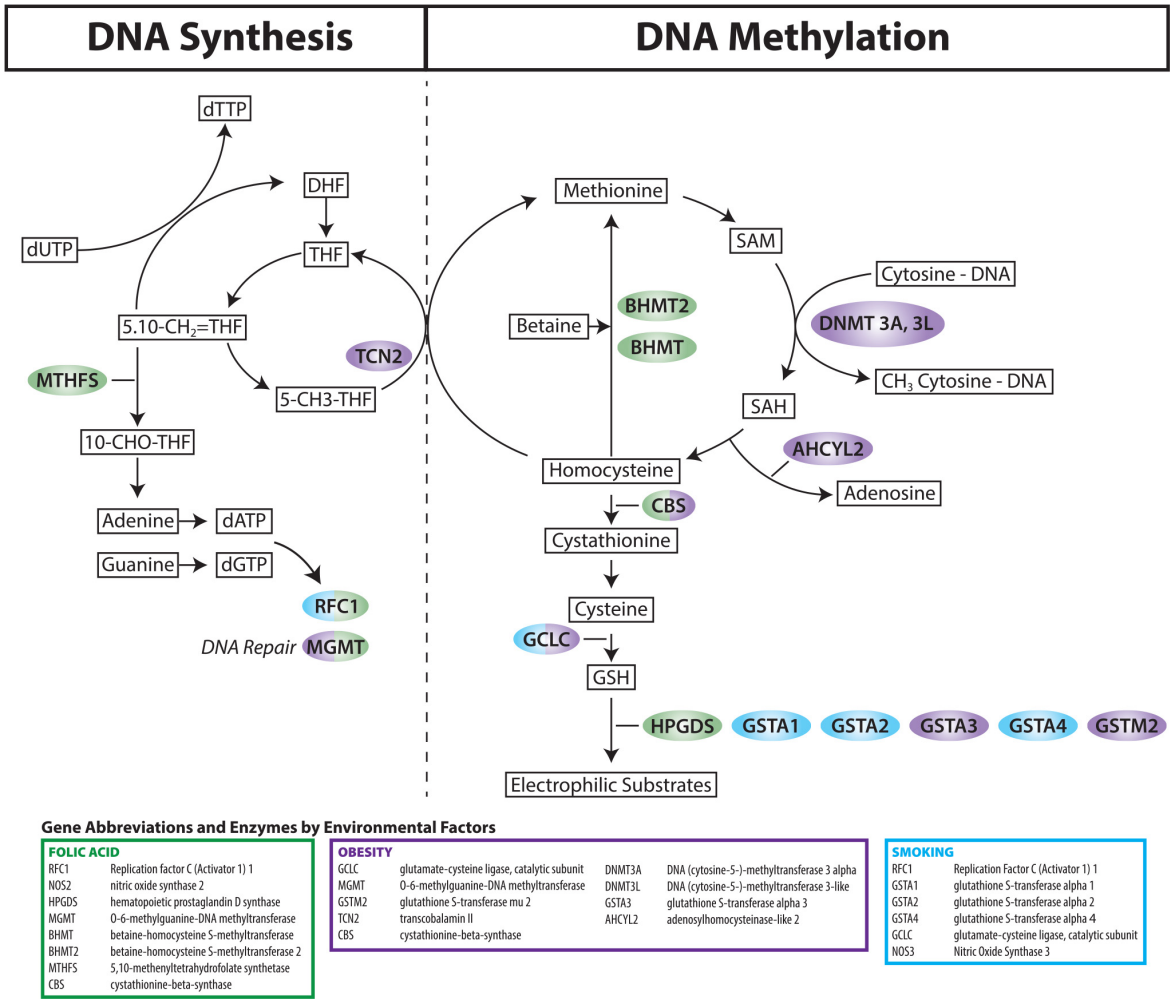
Significant genes and their pathways. Related pathways include the folate pathway, which is involved in DNA synthesis, and the methionine and glutathione pathways, which are necessary for DNA methylation and for conjugation of electrophilic substrates, respectively. Substrates and products of enzymatic reactions appear in boxes. Enzymes appear in color-coded bubbles according to their association with birth defects risk and maternal folic acid supplement use (Green), obesity (Purple) and tobacco use (Blue).

This study provides evidence for a moderating effect of multiple SNPs related to one-carbon metabolism and glutathione metabolism on the relationship between CTDs and maternal obesity and tobacco use. Significant genes and their pathway information are displayed in **Figure 4**. Our findings support the hypothesis that CTDs are associated with G × E interactions between common genetic variants and lifestyle factors. Consistent with previous studies [52]–[54], our results provide supportive evidence that both maternal and fetal genotypes modify the susceptibility of the developing fetus to factors that may alter the *in-utero* environment.

Maternal obesity independent of diabetes is a well-accepted risk factor for multiple nonsyndromic birth defects including CTDs [15], [51], [55]. Among obese women, the maternal genotypes of 10 SNPs within 6 genes (*GCLC, MGMT, GSTM2, TCN2, CBS*, and *DNMT3L*) and the fetal genotypes of 7 SNPs within 3 genes (*GSTA3, AHCYL2*, and *DNMT3A*) were associated with an increased risk of CTDs, whereas, among women of normal weight, none of the maternal genotypes had a significant impact on the RR, and the fetal genotypes of 4 SNPs in 2 genes (*AHCYL2* and *GCLC*) decreased the risk of CTDs.

Among women who used tobacco in the periconceptional period, the maternal genotypes of 6 SNPs in 4 genes (*RFC1, GSTA4, GSTA1*, and *GSTA2*) were associated with an increased risk of CTDs. The *GST* genes function in the detoxification of several environmental exposures, including carcinogens, medications, and factors increasing oxidative stress. The fetal genotypes of 5 SNPs in 3 genes (*GCLC, RFC1* and *NOS3*) increased the occurrence of CTDs among women who smoked. None of the maternal or fetal genotypes significantly impact the risk among women without tobacco use.

Our findings compel us to comment on some of the findings in the context of previous studies. Several maternal and fetal genotypes of SNPs in the glutathione transferase (*GSTA4, GSTA1, GSTA2, GSTM2*, and *GSTA3*) genes increased the impact of maternal obesity and tobacco use on the risk of CTDs. The Glutathione S-transferase complex of genes are involved in the detoxification of a large number of xenobiotics including chemical compounds in tobacco smoke [56]. Genetic variants in glutathione S-transferase genes may be a factor in determining individual susceptibility to embryotoxic effects of xenobiotics, including tobacco. Lending credence to the possibility that glutathione S-transferases in interaction with tobacco may impact the developing fetus, is a report that glutathione S-transferase genetic variants modified the impact of cigarette smoking on birth weight [32].

In earlier reports [57], [58], we demonstrated that women who had infants with CHDs had lower GSH levels. GSH level is essential for cell progression and serves several functions that are crucial during embryogenesis. GSH is important in the defense against oxidative stress, detoxification of xenobiotics, regulation of cell cycle progression and apoptosis, and modulation of immune function [56]. *GCLC* is the gene that encodes for the catalytic subunit glutamate-cysteine ligase, which is the enzyme that catalyzes the conversion of cysteine with glutamate, generating glutathione. Several maternal and fetal genotypes of SNPs in the *GCLC* gene increased the RR of CTDs among women who smoked and/or were obese.

Among women who did not use folic acid supplements, 8 SNPs in the hematopoietic prostaglandin D synthase gene (*HPGDS*) increased the risk of CTDs, but had no associated increased risk among folic acid supplement users [9]. *HPGDS* is a member of the sigma class of glutathione S-transferase gene family. The *HPGDS* gene catalyzes both isomerization of *PGH* (2) to *PGD* (2) and conjugation of glutathione to 1-chlore-2, 4-dinitrobenzene [59]–[61]. *HPGDS* is present in mast cells, macrophages and other cellular sources [62]. Little is known about the impact of prostaglandin synthase on the development of CHDs. Previous studies have shown that *HPGDS* plays a role in multiple physiological processes that are important during embryogenesis, including detoxification of xenobiotics [63], regulation of inflammatory pathways [59] and modification of reactive oxygen species [64]. Animal models and mechanistic studies are needed to validate and clarify the role of maternal *HPGDS* in the development of heart defects.

There are several strengths of this study including the use of log-linear models based on a hybrid design, its large sample size, population-based ascertainment of cases, and a rigorous analytic inquiry into potential G × E interactions on CTD risks. There are also limitations in our study. No adjustment was made for confounding effects from other exposures when fitting the model, which prohibited us from drawing further conclusions about the observed associations. Other limitations related to DNA and lifestyle data collection existed. The usage of buccal cell collection kits in collecting DNA samples created a disparity among the quality of the DNA samples, resulting in a reduction from 1534 to 921 candidate SNPs. Maternal obesity and tobacco use were self-reported, and not objectively measured.

It is now generally accepted that most CHDs are the result of a complex interplay between genetic and environmental factors. Our results suggest that, in the search for genetic variants related to CTDs, accounting for environmental and lifestyle factors may improve the ability to detect genetic effects. Because the typical effect size for maternal and fetal genetic variation acting on CHD phenotypes is small [65], [66], large sample sizes and validation within independent populations are necessary to draw firm conclusions about how certain polymorphisms modulate the experience of maternal lifestyles. The present findings will need to be replicated and further studies should incorporate functional genetics and targeted deep sequencing while enhancing information collected on environmental and lifestyle factors. One limitation of our current study is that to properly assess the accuracy of our findings, they will need to be replicated on a fully distinct and independent sample [67]. Nevertheless, our study is the largest family-based case-control study of CTDs ever conducted in the U.S., and to our knowledge, there is no comparable, independent sample which includes the maternal and fetal genotype data, and maternal lifestyle and demographic data, necessary to validate our results.

## Supporting Information

Table S1 **Maternal and fetal SNPs with interactive effects with maternal obesity.** For each significant SNP, the information about its pathway, chromosome, gene, allele, estimated relative risks and their 95% confidence intervals among normal weight and obese women, p-value and BFDP for the interaction term are presented.
(DOCX)

Table S2 **Maternal and fetal SNPs with interactive effects with maternal tobacco use.** For each significant SNP, the information about its pathway, chromosome, gene, allele, estimated relative risks and their 95% confidence intervals among nonsmokers and smokers, p-value and BFDP for the interaction term are presented.
(DOCX)
