# Supplementary material for: Maternal Obesity and Tobacco Use Modify the Impact of Genetic Variants on the Occurrence of Conotruncal Heart Defects
Source: PLoS One. 2014 Oct 2;9(10):e108903. doi: 10.1371/journal.pone.0108903 (PMC4183535; doi:10.1371/journal.pone.0108903)
Supplement: Table S1 — Maternal and fetal SNPs with interactive effects with maternal obesity. For each significant SNP, the information about its pathway, chromosome, gene, allele, estimated relative risks and their 95% confidence intervals among normal weight and obese women, p-value and BFDP for the interaction term are presented. (DOCX) [file pone.0108903.s001.docx]

**Table S1.** Maternal and fetal SNPs with interactive effects with maternal obesity

| **Maternal effects** | |  |  |  |  |  |  | |
| --- | --- | --- | --- | --- | --- | --- | --- | --- |
|  |  |  |  |  | **Normal weight** | **Obese** | **GXE interaction** | |
| **Chr** | **Gene** | **SNP** | **Pathway** | **Allele*** | **RR (95% CI)** | **RR (95% CI)** | **p-value** | **BFDP^†^** |
| 6 | GCLC | rs6458939 | Glutathione | A/C | 0.89 (0.70, 1.12) | 2.00 (1.41, 2.83) | 1.13×10^-04^ | 0.40 |
| 6 | GCLC | rs742528 | Glutathione | A/T | 0.85 (0.67, 1.07) | 1.81 (1.27, 2.59) | 3.88×10^-04^ | 0.56 |
| 6 | GCLC | rs2397146 | Glutathione | A/G | 0.88 (0.70, 1.11) | 1.84 (1.29, 2.63) | 4.80×10^-04^ | 0.58 |
| 10 | MGMT | rs12219068 | Glutathione | G/A | 0.97 (0.78, 1.22) | 1.97 (1.37, 2.83) | 7.81×10^-04^ | 0.62 |
| 10 | MGMT | rs2039374 | Glutathione | G/A | 1.00 (0.80, 1.25) | 1.94 (1.35, 2.80) | 1.63×10^-03^ | 0.70 |
| 10 | MGMT | rs10764896 | Glutathione | A/G | 0.93 (0.76, 1.14) | 1.55 (1.11, 2.14) | 7.30×10^-03^ | 0.79 |
| 1 | GSTM2 | rs12024479 | Glutathione | G/C | 0.94 (0.76, 1.16) | 1.70 (1.22, 2.36) | 2.04×10^-03^ | 0.69 |
| 22 | TCN2 | rs2301957 | Folate | A/G | 0.89 (0.72, 1.11) | 1.59 (1.15, 2.22) | 3.17×10^-03^ | 0.74 |
| 21 | CBS | rs3972 | Glutathione | A/G | 0.77 (0.57, 1.05) | 1.96 (1.26, 3.06) | 7.33×10^-04^ | 0.75 |
| 21 | DNMT3L | rs2838540 | Homocysteine | A/G | 0.81 (0.63, 1.04) | 1.54 (1.07, 2.21) | 3.25×10^-03^ | 0.77 |
|  |  |  |  |  |  |  |  |  |
| **Fetal effects** | |  |  |  |  |  |  | |
|  |  |  |  |  | **Normal weight** | **Obese** | **GXE interaction** | |
| **Chr** | **Gene** | **SNP** | **Pathway** | **Allele** | **RR (95% CI)** | **RR (95% CI)** | **p-value** | **BFDP^‡^** |
| 6 | GSTA3 | rs668163 | Glutathione | A/G | 0.94 (0.75, 1.18) | 1.83 (1.33, 2.52) | 4.82×10^-04^ | 0.51 |
| 6 | GSTA3 | rs641019 | Glutathione | A/T | 0.99 (0.79, 1.24) | 1.80 (1.31, 2.49) | 1.85×10^-03^ | 0.67 |
| 6 | GSTA3 | rs2281594 | Glutathione | C/G | 0.95 (0.66, 1.36) | 2.56 (1.62, 4.06) | 8.62×10^-04^ | 0.79 |
| 7 | [AHCYL2](http://www.genenames.org/data/hgnc_data.php?hgnc_id=22204) | rs2303303 | Homocysteine | A/G | 0.72 (0.55, 0.93) | 1.52 (1.04, 2.22) | 1.29×10^-03^ | 0.72 |
| 7 | [AHCYL2](http://www.genenames.org/data/hgnc_data.php?hgnc_id=22204) | rs6467233 | Homocysteine | A/G | 0.69 (0.53, 0.89) | 1.39 (0.95, 2.05) | 2.52×10^-03^ | 0.77 |
| 7 | [AHCYL2](http://www.genenames.org/data/hgnc_data.php?hgnc_id=22204) | rs11979476 | Homocysteine | C/A | 0.65 (0.50, 0.85) | 1.29 (0.89, 1.88) | 2.99×10^-03^ | 0.78 |
| 2 | DNMT3A | rs12995245 | Homocysteine | A/G | 0.86 (0.69, 1.07) | 1.48 (1.06, 2.06) | 5.18×10^-03^ | 0.78 |
| 2 | DNMT3A | rs7575625 | Homocysteine | G/A | 0.87 (0.69, 1.08) | 1.49 (1.07, 2.09) | 5.42×10^-03^ | 0.78 |
| 2 | DNMT3A | rs1465825 | Homocysteine | G/A | 0.85 (0.66, 1.10) | 1.59 (1.11, 2.29) | 4.28×10^-03^ | 0.79 |
| 6 | GCLC | rs2066511 | Glutathione | A/G | 0.74 (0.57, 0.97) | 1.47 (0.99, 2.16) | 3.56×10^-03^ | 0.79 |

Chr: chromosome; RR: relative risk; CI: confidence interval

* Allele is presented as minor/major allele in our study sample; major allele is the reference allele.

**^†^** Interactive BFDP was based on the test for the hypothesis whether there was a significant interaction between maternal SNP and obesity on the risk of disease.

^‡^ Interactive BFDP was based on the test for the hypothesis there was a significant interaction between fetal SNP and maternal obesity on the risk of disease.
