# Supplementary material for: Maternal Obesity and Tobacco Use Modify the Impact of Genetic Variants on the Occurrence of Conotruncal Heart Defects
Source: PLoS One. 2014 Oct 2;9(10):e108903. doi: 10.1371/journal.pone.0108903 (PMC4183535; doi:10.1371/journal.pone.0108903)
Supplement: Table S2 — Maternal and fetal SNPs with interactive effects with maternal tobacco use. For each significant SNP, the information about its pathway, chromosome, gene, allele, estimated relative risks and their 95% confidence intervals among nonsmokers and smokers, p-value and BFDP for the interaction term are presented. (DOCX) [file pone.0108903.s002.docx]

**Table S2.** Maternal and fetal SNPs with interactive effects with maternal tobacco use

| **Maternal effects** | | |  |  |  |  |  | |
| --- | --- | --- | --- | --- | --- | --- | --- | --- |
|  |  |  |  |  | **Nonsmokers** | **Smokers** | **GXE interaction** | |
| **Chr** | **Gene** | **SNP** | **Pathway** | **Allele^*^** | **RR (95% CI)** | **RR (95% CI)** | **p-value** | **BFDP^†^** |
| 4 | RFC1 | rs13123782 | DNA Synthesis/repair | A/C | 0.94 (0.79, 1.11) | 1.60 (1.18, 2.18) | 1.92×10^-03^ | 0.64 |
| 4 | RFC1 | rs6531712 | DNA Synthesis/repair | T/A | 0.91 (0.77, 1.08) | 1.49 (1.08, 2.05) | 5.47×10^-03^ | 0.76 |
| 6 | GSTA4 | rs2397135 | Glutathione | A/C | 0.92 (0.76, 1.11) | 1.74 (1.21, 2.49) | 1.68×10^-03^ | 0.68 |
| 6 | GSTA4 | rs182623 | Glutathione | A/T | 0.95 (0.79, 1.14) | 1.70 (1.19, 2.43) | 3.63×10^-03^ | 0.76 |
| 6 | GSTA1 | rs9474321 | Glutathione | G/A | 0.91 (0.76, 1.08) | 1.51 (1.10, 2.08) | 5.06×10^-03^ | 0.76 |
| 6 | GSTA2 | rs4715318 | Glutathione | A/G | 0.88 (0.73, 1.06) | 1.50 (1.07, 2.08) | 4.65×10^-03^ | 0.76 |
|  |  |  |  |  |  |  |  |  |
| **Fetal effects** | | |  |  |  |  |  |  |
|  |  |  |  |  | **Nonsmokers** | **Smokers** | **GXE interaction** | |
| **Chr** | **Gene** | **SNP** | **Pathway** | **Allele** | **RR (95% CI)** | **RR (95% CI)** | **p-value** | **BFDP^‡^** |
| 6 | GCLC | rs7742367 | Glutathione | G/A | 1.14 (0.93, 1.40) | 2.12 (1.48, 3.04) | 2.15×10^-03^ | 0.71 |
| 6 | GCLC | rs10948751 | Glutathione | C/A | 1.15 (0.94, 1.41) | 2.03 (1.42, 2.91) | 4.69×10^-03^ | 0.78 |
| 6 | GCLC | rs2284650 | Glutathione | G/A | 0.83 (0.61, 1.12) | 2.58 (1.47, 4.53) | 4.30×10^-04^ | 0.79 |
| 4 | RFC1 | rs11727502 | DNA Synthesis/repair | C/A | 0.90 (0.76, 1.08) | 1.50 (1.08, 2.08) | 5.27×10^-03^ | 0.76 |
| 7 | NOS3 | rs10277237 | Glutathione | A/G | 0.91 (0.74, 1.11) | 1.59 (1.11, 2.29) | 5.70×10^-03^ | 0.79 |

Chr: chromosome; RR: relative risk; CI: confidence interval

* Allele is presented as minor/major allele in our study sample; major allele is the reference allele.

**^†^** Interactive BFDP was based on the test for the hypothesis whether there was a significant interaction between maternal SNP and tobacco use on the risk of disease.

^‡^ Interactive BFDP was based on the test for the hypothesis there was a significant interaction between fetal SNP and maternal tobacco use on the risk of disease.
